# Ideal cardiovascular health and all-cause or cardiovascular mortality in a longitudinal study of the Thai National Health Examination Survey IV and V

**DOI:** 10.1038/s41598-023-29959-1

**Published:** 2023-02-16

**Authors:** Wichai Aekplakorn, Nareemarn Neelapaichit, Suwat Chariyalertsak, Pattapong Kessomboon, Sawitri Assanangkornchai, Surasak Taneepanichskul, Somkiat Sangwatanaroj, Wasin Laohavinij, Jiraluck Nonthaluck

**Affiliations:** 1 Department of Community Medicine, Faculty of Medicine Ramathibodi Hospital, Mahidol University, Rama VI Rd., Ratchathewi, Bangkok, Thailand; 2 Ramathibodi School of Nursing, Faculty of Medicine, Ramathibodi Hospital, Mahidol University, Bangkok, Thailand; 3 Faculty of Public Health, Chiang Mai University, Chiang Mai, Thailand; 4 Faculty of Medicine, Khon Kaen University, Khon Kaen, Thailand; 5 Epidemiology Unit, Faculty of Medicine, Prince of Songkla University, Songkhla, Thailand; 6 College of Public Health Sciences, Chulalongkorn University, Bangkok, Thailand; 7 Department of Internal Medicine, Faculty of Medicine Chulalongkorn Hospital, Chulalongkorn University, Rama VI Rd., Pathumwan, Bangkok, Thailand; 8 Department of Preventive and Social Medicine, Faculty of Medicine, Chulalongkorn University, Bangkok, Thailand; 9 Department of Epidemiology, Faculty of Public Health, Mahidol University, Bangkok, Thailand

**Keywords:** Health care, Medical research, Risk factors

## Abstract

The relationship of ideal cardiovascular health (CVH) and health outcomes has been rarely assessed in middle-income countries. We determined the ideal CVH metrics and association with all-cause and cardiovascular (CVD) mortality in the Thai population. We used baseline data from two rounds of the National Health Examination survey (15,219 participants in 2009 and 14,499 in 2014), and assessed all-cause and CVD deaths until 2020. The prevalence of 5–7 ideal CVH metrics in 2009 was 10.4% versus 9.5% in 2014. During a median follow-up of 7.1 years, the all-cause and CVD mortality rates were 19.4 and 4.6 per 1000 person-years for 0–1 ideal CVH metrics, and 13.0 and 2.1, 9.6 and 1.5, 6.0 and 1.0, and 2.9 and 0.4 per 1000 person-years for 2, 3, 4, and 5–7 ideal CVH metrics, respectively. Participants with 2, 3, 4, or 5–7 ideal metrics had a significantly lower risk of mortality than those with 0–1 ideal CVH metrics (adjusted hazard ratios: 0.75, 0.70, 0.60, and 0.47 for all-cause, and 0.54, 0.52, 0.50, and 0.31 for CVD, respectively). Individuals with a higher number of the modified ideal CVH metrics have a lower risk of all-cause and CVD mortality.

## Introduction

Noncommunicable diseases (NCDs) cause 41 million deaths each year worldwide and account for 71% of total deaths. The majority (80%) of this burden occurs in low- and middle-income countries, and cardiovascular disease (CVD) is the leading cause of death^1^. The World Health Organization set a global NCD monitoring framework for NCDs in each country with the main target of a 25% reduction in mortality for NCD outcomes in 2030^2^. The American Heart Association (AHA)’s Life Simple 7 (LS7) was introduced to assess the extent of ideal cardiovascular health (CVH) at the population level^3^. Several studies have shown that the metabolic and behavioral factors in the LS7, which comprises blood pressure, weight (body mass index [BMI]), smoking, diet, physical activity, blood sugar, and the total cholesterol concentration, are associated with all-cause and CVD mortality^4^. These metrics have been applied and assessed in the US population, Europe, and some other countries, such as China and Korea, but these countries have different CVH metrics and estimates^5,6^. Additionally, these metrics have been rarely assessed in the southeast Asian and low- and middle-income country populations, where the population characteristics and the availability of metrics to be measured might be different. Some metrics were based on local studies with varied strengths of associations. A systematic review reported that the waist-to-height ratio (WHtR) had a better discriminatory power than BMI to predict cardiometabolic health outcome and mortality^7^. Studies in the Thai population showed that central obesity, especially the WHtR, was a better risk factor for atherosclerotic CVD than BMI^8^. Serum high-density-lipoprotein cholesterol (HDL-C) concentrations were better associated with all-cause, CVD, and cancer mortality than serum total cholesterol concentrations in a Thai cohort study^9^.

The Thai National Health Examination Survey (NHES) showed an increasing prevalence of obesity, diabetes, and hypertension in the Thai population in the most recent 2 decades^10–12^. These data provided an opportunity to follow-up these diseases and examine their association with mortality. This study aimed to determine the national prevalence of ideal CVH metrics in Thai adults and to examine the association of modified CVH metrics with all-cause and CVD mortality.

## Methods

### Study population

This study analyzed the data from the NHESs IV and V, which were conducted in 2009 and in 2014. The baseline data for participants were followed up until October 2020 and merged with mortality data from the vital statistics. The NHES is a multi-stage, nationally representative sampled survey of the Thai population, and it is a periodic national health examination survey. This survey uses a complex multi-stage probability sampling design to represent the national Thai population aged ≥ 1 year. The NHESs obtain data on a range of sociodemographics, health behavior, and health status through interviews, anthropometric measurements of body weight (kg), height, and waist circumference (cm), blood pressure (BP) measurement (mmHg), and blood samples. The blood samples were used to measure serum glucose and cholesterol concentrations. Details of the survey were described elsewhere^8^. The present study used the data collected in 2009 and 2014 for participants aged 20 years and older. The primary outcome of the study was all-cause mortality for all participants of the 2009 and 2014 cohort. The secondary outcome was cause-specific mortality of CVD only in the cohort in 2014 because of the availability of electronic health records for ascertaining outcomes. Ethical approval for this study was obtained from the Human Research Ethics Committee, Faculty of Medicine Ramathibodi Hospital, Mahidol University (COA. MURA2021/551). The Ethical Review is based on the foundation ethical principles embodied in accordance with the Declaration of Helsinki of 1964 and its subsequent revision and the Belmont Report. Written informed consent was obtained from all participants during the initial assessment. All methods were performed in accordance with the relevant guidelines and regulations.

### Definitions

Table 1 shows the modified definitions of CVH metrics. Collected baseline data were age, sex, area of residence (urban/rural), smoking (based on the self-reported by questionnaire interview), physical activity level (according to the global physical activity questionnaire of the World Health Organization), waist-to-height ratio (waist circumference was measured using tape placed horizontally around the waist at a mid-point between the anterior iliac crest and lower rib; height was measured using a stadiometer), serum HDL-C concentrations (mg/dL, based on laboratory measurement), fruit and vegetable consumption (portions/day, based on self-report by questionnaire interview), fasting plasma glucose (based on fasting plasma glucose measurements and a history of physician-diagnosed diabetes and taking an reducing hyperglycemic drug), and hypertension (based on blood pressure measurements and a history of physician-diagnosed hypertension and taking an antihypertensive drug). The total ideal CVH was considered as the total number of ideal CVH metrics and categorized into 0–1, 2, 3, 4, and 5–7 ideal CVH metrics. We also calculated an overall CVH score ranging from 0 (poorest) to 14 (most ideal), in which individual metrics were defined as 0 (poor), 1(intermediate) and 2 (ideal). The overall score was divided into 3 groups based on the tertile of overall CVH score; inadequate = 0–6, average = 7–8, and optimal = 9–14.

**Table 1:** Cardiovascular health (CVH) metrics.

	2 = Ideal	1 = Intermediate	0 = Poor
Smoking	Non-smokers	Ex-smokers	Current smokers
Physical activity	≥ 150 min/week moderate physical activity, ≥ 75 min/week vigorous physical activity, or ≥ 150 min/week combined intensity	1–149 min/week moderate physical activity, 1–74 min/week vigorous exercise, or 1–149 min/week combined intensity	0 to < 1 min/week moderate physical activity, vigorous physical activity, or combined intensity
Obesity indicated by the WHtR	≤ 0.5	> 0.5 and < 0.6	≥ 0.6
Dietary intake indicated by fruit and vegetable consumption	Fruit and vegetable consumption of > 5 portions/day	Fruit and vegetable consumption of 3–5 portions/day	Fruit and vegetable consumption of < 3 portions/day
Dyslipidemia indicated by low HDL-C concentrations	≥ 60 mg/dL	40 to < 60 mg/dL in men	< 40 mg/dL in men
		50 to < 60 mg/dL in women	and < 50 mg/dL in women
Fasting plasma glucose	< 100 mg/dL	100–125.9 mg/dL and/or diabetes with treatment	≥ 126 mg/dL and/or diabetes
Hypertension	SBP < 120 and DBP < 80 mmHg	SBP of 120–139 mmHg, DBP of 80–89 mmHg, or treated to goal	SBP ≥ 140 or DBP ≥ 90 mmHg

WHtR, waist-to-height ratio; HDL-C, high-density lipoprotein cholesterol; SBP, systolic blood pressure; DBP, diastolic blood pressure.

### Outcome assessment

The primary outcome of this study was all-cause mortality for the NHES IV and V. The secondary outcome was cause-specific CVD mortality for the NHES V because ascertainment of the underlying cause of death in the electronic medical records has been available since 2014, but it is not available for the NHES IV. The causes of death were obtained from death certificates and review of medical record discharges with electronic health records from the National Health Security Office, and were verified and adjudicated by a committee (the authors). The causes of CVD death were identified according to the International Statistical Classification of Diseases and Related Health Problems, Tenth Revision, including heart diseases, ischemic heart diseases, and cerebrovascular diseases (I00-I09, I11, I13 I20-I51, and I60-I69).

### Statistical analysis

We included participants with a BMI ≥ 18.5 kg/m^2^ in the analysis. Participants with a BMI < 18.5 kg/m^2^ associated with increased mortality^14^, and those with a history of a heart attack, myocardial infarction, stroke, or chronic kidney disease (stage ≥ 3) were excluded from the analysis. The probability of sampling weights was used in the analyses. All of the analyses were weighted to the probability of sampling. The weighted prevalence and 95% confidence interval (CI) of seven simple metrics were calculated. At baseline, the prevalence of each CVH metric and the numbers of ideal CVH metrics and behavior factors were computed overall and in subgroups in the 2009 and 2014 surveys and compared using Chi-squared test. The subgroups comprised sex and age subgroups (20–39, 40–59, and ≥ 60 years). With regard to the outcome after the follow-up, the number of person-years was counted from baseline until the date of death, loss to follow-up, or September 2020. Cumulative incidence function curves for all-cause mortality and CVD mortality by the number of ideal CVH metrics were created and tested for significance using the log-rank test. Cox proportional hazard regression was used to examine the associations between outcome variables and the number of ideal CVH metrics controlling for age (20–39, 40–59, and ≥ 60 years) and sex in model 1. In the fully adjusted model, the educational level (primary, secondary, and university), area of residence (urban/rural), alcohol consumption (based beverage-specific and graduated quantity-frequency questionnaire to measure the amount of alcohol consumed and categorized into abstainer, low risk, medium risk, and high risk)^,^^13^ and dyslipidemia medication were included. Hazard ratios (HRs) and 95% CIs were reported. Proportional hazards assumptions were evaluated by testing time-dependent interaction terms, and we did not observe violation of the assumption. HRs were calculated according to the number of ideal factors (0–1 (reference), 2, 3, 4, 5–7 and 0–1, 2–4, and 5–7 categories and the overall CVH score as a continuous variable; a categorical variable (0–6 = inadequate (reference), 7–8 = average, and 9–14 = optimal). The data for the NHES V were able to be used for the analysis of the association of CVD death. The cause of death was verified by electronic medical records from the National Health Security Office. When the NHES IV was conducted in 2009, the electronic records were not completely available. Consequently, we were not able to ascertain CVD death in the NHES IV cohort. Stata program version 16 (StataCorp, College Station, TX, USA) was used for all analyses, and statistical significance was set at P < 0.05 with two-tailed tests.

## Results

A total of 29,718 adults (15,219 from the NHES IV in 2009 and 14,499 from the NHES V in 2014) (men: 13,001, women: 16,717) were included in the analyses between 2009 and 2020. Table 2 shows the mean (SD) age was 45.4 (13.6) years in 2009 and 46.8 (15.0) years in 2014. There were 3015 deaths with a total of 268,766.2 person-years and a mean (SD) follow-up of 10.9 (2.4) years (median: 7.1 years). Of the 722 deaths in the 2014 cohort, 221 were caused by CVD.

**Table 2:** Baseline characteristics and weighted prevalence of ideal CVH metrics by sex in adults from the NHES IV in 2009 and in the NHES V in 2014.

	NHES IV				NHES V				P-value*
	Number	Male	Female	Total	Number	Male	Female	Total	
Mean age (years)	15,219	44.7 (43.6, 45.9)	46.1 (45.3, 46.8)	45.4 (44.5, 46.4)	14,499	46.1 (44.9, 47.2)	47.5 (46.7, 48.3)	46.8 (45.9, 47.8)	0.09
Age group (years)									
20–39	3377	36.2 (32.4, 40.1)	33.1 (30.7, 35.6)	34.6 (31.6, 37.6)	2668	34.5 (30.8, 38.3)	31.3 (29.1, 33.6)	32.8 (30.0, 35.7)	0.01
40–59	5181	51.1 (48.5, 53.6)	51.8 (49.1, 54.7)	51.4 (49.1, 53.7)	6277	48.1 (45.0, 51.2)	48.3 (46.0, 50.6)	48.2 (45.9, 50.6)	
≥ 60	6661	12.8 (11.0, 14.8)	15.1 (13.6, 16.7)	14.0 (12.4, 15.8)	5554	17.4 (15.6, 19.3)	20.4 (18.4, 22.7)	19.0 (17.2, 21.0)	
Sex									
Female	8142	–	–	51.9 (50.2, 53.7)	8575	–	–	53.1 (52.0, 54.3)	0.21
Area of residence									
Urban	8142	29.2 (17.7, 44.2)	31.6 (19.3, 47.2)	30.5 (18.5, 45.8)	7713	43.8 (28.8, 60.1)	45.8 (30.0, 62.6)	44.9 (29.4, 61.4)	< 0.05
Education									
No formal	909	1.8 (1.2, 2.6)	5.1 (4.1, 6.3)	3.5 (2.8, 4.4)	667	1.9 (1.2, 3.0)	4.1 (3.1, 5.3)	3.1 (2.3, 4.1)	< 0.05
Primary	9623	59.4 (54.8, 63.8)	65.5 (61.3, 69.4)	62.5 (58.4, 66.5)	8742	51.7 (44.2, 59.2)	57.1 (52.0, 62.0)	54.6 (48.4, 60.6)	
Secondary	3669	32.6 (29.8, 35.7)	22.7 (19.7, 26.0)	27.5 (24.8, 30.3)	3678	35.0 (31.4, 38.9)	28.6 (26.1, 31.2)	31.6 (28.8, 34.5)	
University	1018	6.2 (4.8, 8.1)	6.7 (5.4, 8.3)	6.5 (5.2, 8.1)	1412	11.3 (6.9, 18.0)	10.3 (7.1, 14.7)	10.8 (7.0, 16.2)	
Alcohol consumption									
Abstain and low	14,019	84.3 (81.5, 86.6)	97.0 (96.1, 97.7)	90.9 (89.6, 92.0)	13,914	90.7 (88.7, 92.4)	98.2 (97.5, 98.7)	94.7 (93.5, 95.7)	< 0.05
Medium to high	1192	15.7 (13.40	3.0 (2.3, 3.9)	9.1 (8.0, 10.4)	585	9.3 (7.6, 11.3)	1.8 (1.3, 2.5)	5.3 (4.3, 6.5)	
CVH metrics									
Smoking									
Never	9898	31.8 (28.6, 35.3)	95.2 (93.7, 96.4)	64.8 (62.3, 67.1)	9965	35.6 (31.4, 40.1)	92.8 (90.8, 94.4)	66.0 (63.4, 68.5)	< 0.001
Former	2173	20.8 (17.9, 23.9)	1.9 (1.3, 2.7)	11.0 (9.4, 12.7)	2174	25.3 (23.6, 27.2)	4.7 (3.5, 6.1)	14.3 (13.4, 15.3)	
Current	3148	47.4 (43.7, 51.2)	2.8 (2.0, 3.9)	24.3 (22.3, 26.4)	2360	39.1 (34.6, 43.8)	2.5 (1.8, 3.5)	19.7 (17.3, 22.2)	
Waist-to-height ratio									
Ideal (< 0.5)	6450	56.0 (51.0, 60.9)	38.9 (34.7, 43.2)	47.1 (43.0, 51.3)	4870	48.7 (44.5, 52.9)	31.9 (29.8, 34.0)	39.7 (37.0, 42.6)	< 0.001
Intermediate (0.5 to < 0.6)	6948	37.8 (34.1, 41.6)	47.3 (44.7, 50.0)	42.8 (40.1, 45.4)	7058	42.7 (39.0, 46.5)	47.6 (45.9, 49.3)	45.3 (43.2, 47.4)	
Poor (≥ 0.6)	1821	6.2 (4.7, 8.1)	13.8 (11.7, 16.2)	10.1 (8.4, 12.2)	2559	8.6 (7.7, 9.6)	20.6 (19.2, 22.1)	15.2 (14.0, 16.1)	
Vegetables and fruits									
Ideal	4250	27.6 (25.5, 29.9)	30.5 (28.0, 33.2)	29.1 (27.1, 31.3)	3422	23.3 (18.5, 29.0)	26.1 (23.1, 29.3)	24.8 (21.0, 29.0)	0.08
Intermediate	3962	26.0 (24.6, 27.5)	26.0 (24.4, 27.7)	26.0 (24.8, 27.3)	4259	28.5 (26.6, 30.6)	30.4 (27.7, 33.3)	29.5 (27.3, 31.9)	
Poor	7007	46.4 (43.8, 49.0)	43.4 (40.4, 46.5)	44.9 (42.4, 47.4)	6818	48.2 (42.7, 53.6)	43.5 (39.3, 47.8)	45.7 (41.0, 50.4)	
Physical activity									
Ideal	1694	15.8 (14.1, 17.6)	8.4 (7.5, 9.5)	12.0 (10.8, 13.2)	841	10.0 (8.7, 11.4)	4.6 (3.4, 6.2)	7.1 (6.1, 8.4)	< 0.001
Intermediate	1641	9.5 (8.6, 10.4)	9.9 (8.9, 11.1)	9.7 (9.2, 10.3)	1915	10.9 (9.7, 12.2)	11.2 (9.8, 12.7)	11.1 (10.1, 12.1)	
Poor	11,884	74.8 (72.6, 76.8)	81.6 (79.8, 83.3)	78.3 (76.8, 79.8)	11,743	79.1 (77.4, 80.7)	84.2 (81.7, 86.4)	81.8 (80.0, 83.5)	
BP (mmHg)									
(SBP/DBP < 120/80, untreated)	5144	37.6 (32.7, 42.8)	47.9 (45.9, 49.9)	42.9 (40.0, 46.0)	4729	33.2 (30.5, 35.9)	46.0 (41.8, 50.2)	40.0 (36.8, 43.2)	0.10
Intermediate (SBP of 120–129, DBP < 80, or treated to goal)	5204	40.8 (38.2, 43.4)	31.2 (29.6, 32.9)	35.8 (34.0, 37.7)	4929	40.6 (38.9, 42.3)	29.7 (28.0, 31.4)	34.8 (33.4, 36.2)	
SBP ≥ 130 or DBP ≥ 80	4871	21.7 (18.2, 25.5)	20.9 (19.1, 22.7)	21.2 (18.9, 23.8)	4841	26.2 (24.1, 28.6)	24.4 (21.7, 27.3)	25.3 (23.3, 27.3)	
HDL-C (mg/dL)	15,219				14,499				
Ideal (≥ 60)	1956	9.1 (7.4, 11.3)	15.0 (11.8, 18.9)	12.2 (9.7, 15.2)	3010	15.5 (12.7, 18.7)	23.4 (21.0, 25.8)	19.6 (17.3, 22.2)	< 0.01
Intermediate (40/50 for male/female to < 60)	5926	54.9 (52.5, 57.1)	25.1 (23.6, 26.7)	39.4.2 (38.3, 40.6)	5298	51.4 (49.1, 53.7)	26.4 (25.0, 27.8)	38.1 (36.8, 39.4)	
Poor (< 40 for male and < 50 for female)	7337	36.0 (32.5, 39.7)	59.9 (55.3, 64.3)	48.4 (45.0, 51.8)	6191	33.1 (29.3, 37.2)	50.3 (47.1, 53.5)	42.3 (39.1, 45.5)	
FPG (mg/dL)	15,219				14,499				
Ideal (< 100)	11,541	81.4 (77.0, 85.1)	81.8 (78.9, 84.5)	81.6 (78.2, 84.6)	10,113	73.0 (69.4, 76.3)	74.1 (70.9, 77.0)	73.6 (70.5, 76.4)	< 0.001
Intermediate (100–125)	2006	12.3 (9.4, 16.0)	9.8 (7.3, 13.1)	11.0 (8.4, 14.4)	2568	18.3 (15.6, 21.4)	15.5 (13.2, 18.2)	16.8 (14.6, 19.3)	
Poor (≥ 126)	1672	6.3 (5.2, 7.6)	8.3 (7.2, 9.6)	7.4 (6.6, 8.2)	1818	8.7 (7.3, 10.3)	10.4 (9.3, 11.6)	9.6 (8.5, 10.8)	

Data presented as mean (95%CI) and prevalence (%, 95%CI).

*P-value for Chi-squared test compare total prevalence between NHES IV and NHES V.

The baseline weighted prevalence of CVH metrics in 2009 and 2014 were as follows: non-smoking, 64.8% and 65.7%; ideal WHtR, 49.7 and 39.5; ideal vegetable and fruit consumption, 29.1%, and 24.9%; ideal physical activity level, 78.4%, and 81.9%; ideal blood pressure, 42.7% and 39.7%; ideal HDL-C concentrations, 12.2%, and 19.7%; and ideal fasting plasma glucose concentrations, 81.7%, and 73.4%, respectively (Table 2).

Table 3 shows that the prevalence of having 4 and 5–7 ideal CVH metrics in 2014 was slightly lower than that in 2009. The prevalence of 4 and 5–7 ideal CVH metrics in 2009 was 21.2% and 10.4% versus 17.9% and 9.5% in 2014, respectively. The prevalence of individuals who achieved 5–7 ideal CVH metrics were much lower in men than in women (6% vs 14.4% in 2009 and 5.6% vs 12.9% in 2014, respectively). For overall CVH score category, compared to 2009, the prevalence of inadequate CVH score category (0–6) increased in 2014, while the prevalence of optimal CVH score category (9–14) decreased in 2014.

**Table 3:** Baseline weighted prevalence (95% CI) of number of ideal CVH metrics by sex from the NHES IV in 2009 and the NHES V in 2014.

	NHES IV				NHES V				
	Number	Male	Female	Total	Number	Male	Female	Total	P -value
Number of ideal CVH metrics	15,219				14,499				
0–1	2789	19.5 (16.9, 22.4)	8.4 (7.2, 9.6)	13.7 (12.0, 15.6)	3176	26.2 (23.8, 28.9)	12.1 (10.6, 13.8)	18.7 (17.1, 20.5)	< 0.001
2	4254	27.7 (25.7, 29.8)	22.4 920.8, 24.1)	25.0 (23.7, 26.3)	4284	28.7 (26.5, 31.1)	25.8 (24.0, 27.6)	27.2 (25.5, 28.9)	
3	4167	29.9 (27.6, 32.3)	29.6 (27.9, 31.4)	29.8 (28.3, 31.2)	3727	25.1 (23.5, 26.8)	28.3 (26.2, 30.5)	26.8 (25.5, 28.1)	
4	2686	16.9 (15.9, 18.0)	25.2 (23.5, 27.0)	21.2 (20.4, 22.1)	2188	14.3 (13.0, 15.8)	21.0 (19.3, 22.7)	17.9 (16.6, 19.2)	
5–7	1323	6.0 (5.1, 6.9)	14.4 (12.7, 16.3)	10.4 (9.2, 11.6)	1124	5.6 (4.7, 6.7)	12.9 (11.4, 14.5)	9.5 (8.6, 10.4)	
Overall CVH score									
Inadequate (0–6)	5714	35.7 (33.1, 38.5)	25.6 (23.6, 27.6)	30.5 (28.5, 32.5)	5966	42.1 (39.6, 44.6)	30.8 (28.6, 33.1)	36.1 (34.2, 38.1)	< 0.001
Average (7–8)	5161	37.3 (35.4, 39.2)	35.0 (33.1, 37.0)	36.1 (34.6, 37.6)	4785	33.9 (31.6, 36.3)	33.6 (32.3, 34.8)	33.7 (32.4, 35.1)	
Optimal (9–14)	4344	27.0(25.3, 28.7)	39.4 (37.1, 41.8)	33.4 (31.8, 35.1)	3748	24.0 (21.7, 26.5)	35.6 (33.3, 37.9)	30.2 (28.1, 32.3)	

*P-value for Chi-squared test.

### Association of CVH metrics and all-cause mortality

Table 4 shows the mortality rate according to modified CVH metrics and associated HRs. There were 3108 all-cause deaths from 2009 to 2020, with a mortality rate of 11.2 per 1000 person-years. The crude mortality rate decreased as the number of ideal CVH metrics increased as follows: 0–1, 2, 3, 4, and 5–7 ideal CVH metrics were associated with 19.4, 13.0, 9.6, 6.0, and 2.9 per 1000 person-years. Two hundred twenty-two individuals died from CVD causes, with a mortality rate of 2.2 per 1000 person-years during follow-up time of 7.1 years. The CVD mortality rate decreased as the number of ideal CVH metrics increased, with a corresponding mortality rate of 4.6, 2.1, 1.5, 1.0, and 0.4 per 1000 person-years, respectively. After adjusted for potential confounders, the HRs for all-cause mortality for 2, 3, 4, and 5–7 ideal CVH metrics were 0.75, 0.70, 0.60, and 0.47, respectively, compared with 0–1 ideal CVH metrics. The corresponding HRs for CVD death were 0.54, 0.52, 0.50, and 0.31, respectively. Analyses using an overall CVH score showed similar results. A unit increase in overall CVH score was significantly associated with 12% and 19% lower risk of all-cause mortality and CVD mortality, respectively. Compared with individuals in inadequate CVH score category (0–6), the adjusted HRs for all-cause mortality for those who achieved optimal CVH score (9–14) and average CVH score (7–8), were 0.53 and 0.70, respectively. The corresponding HRs for CVD death were 0.51, and 0.53, respectively.

**Table 4:** All-cause and CVD mortality rate (per 1000 person-years) and Hazard Ratios (HR) by ideal CVH category.

Number of ideal CVH metrics	All-cause					CVD				
	No. of deaths	Person-years	Mortality rate	Age-adjusted HR* (95% CI)	Fully adjusted HR** (95% CI)	No. of deaths	Person-years	Mortality rate	Age-adjusted HR* (95% CI)	Fully adjusted HR** (95% CI)
0–1	988	50,992.2	19.4 (18.2, 20.6)	1	1	101	21 804.5	4.6 (3.8, 5.6)	1	1
2	987	75,889.6	13.0 (12.2, 13.8)	0.76 (0.60, 0.95)	0.75 (0.59, 0.94)	63	29 650.9	2.1 (1.7, 2.7)	0.54 (0.39, 0.75)	0.54 (0.39, 0.74)
3	696	72,495.6	9.6 (8.9, 10.4)	0.71 (0.57, 0.89)	0.70 (0.56, 0.88)	38	26 033.4	1.5 (1.1, 2.0)	0.50 (0.34, 0.74)	0.52 (0.35, 0.74)
4	277	46,044.3	6.0 (5.3, 6.8)	0.61 (0.44, 0.83)	0.60 (0.43, 0.82)	16	15 367.8	1.0 (0.6, 1.7)	0.49 (0.29, 0.85)	0.50 (0.30, 0.90)
5–7	67	23,344.5	2.9 (2.3, 3.7)	0.46 (0.30, 0.69)	0.47 (0.30, 0.73)	3	7980.4	0.4 (0.1, 1.2)	0.28 (0.09, 0.93)	0.31 (0.09, 1.04)
Total	3015	268,766.5	11.2 (10.8, 11.6)	–	–	221	100 733.7	2.2 (1.9, 2.5)	–	
Overall CVH score category										
Inadequate (0–6)	1774	101,474.97	17.5 (16.7, 18.3)	1	1	155	41,030.10	3.8 (3.2, 4.4)	1	1
Average (7–8)	876	90,971.58	9.6 (9.0, 10.3)	0.70 (0.59, 0.82)	0.70 (059, 0.82)	45	33,362.99	1.4 (1.0, 1.8)	0.51 (0.37, 0.71)	0.53 (0.38, 0.74)
Optimal (9–14)	365	76,319.65	4.8 (4.3, 5.3)	052 (0.41, 0.66)	0.53 (0.42, 0.68)	21	26,340.58	0.8 (0.5, 1.2)	0.46 (0.29, 0.74)	0.51 (0.32, 0.82)
Overall CVH score increase per unit)		–	–	0.88 (0.84, 0.92)	0.88 (0.84, 0.92)		–	–	0.80 (0.74, 0.86)	0.81 (0.75, 0.87)

*Adjusted for age and sex; ** adjusted for age, sex, education, urban/rural area, and alcohol drinking and cholesterol drug medication.

**Figure 1:**
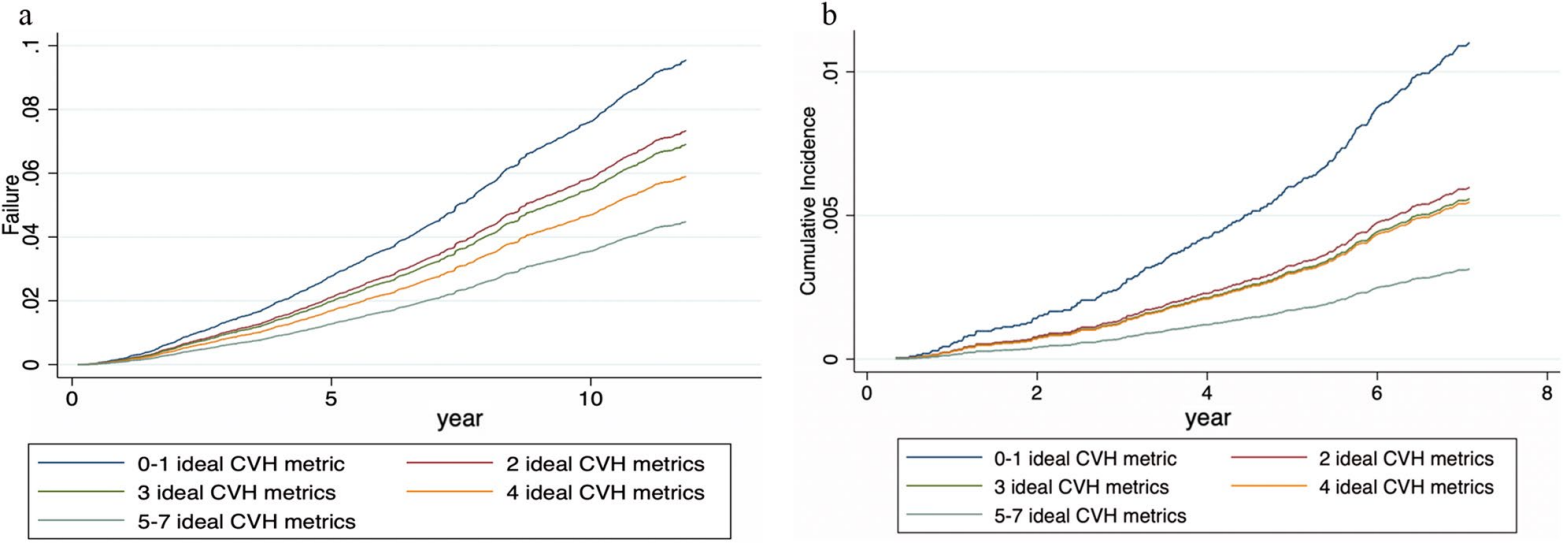
(**a**) Cumulative function curve for all-cause mortality by ideal CVH metrics (blue line: 0–1; red: 2; dark green: 3; orange: 4, and light green: 5–7 ideal CVH metrics). (**b**) Cumulative function curve for CVD mortality by ideal CVH metrics (blue line:0–1; red: 2; dark green: 3; orange: 4, and light green: 5–7 ideal CVH metrics).

**Figure 2:**
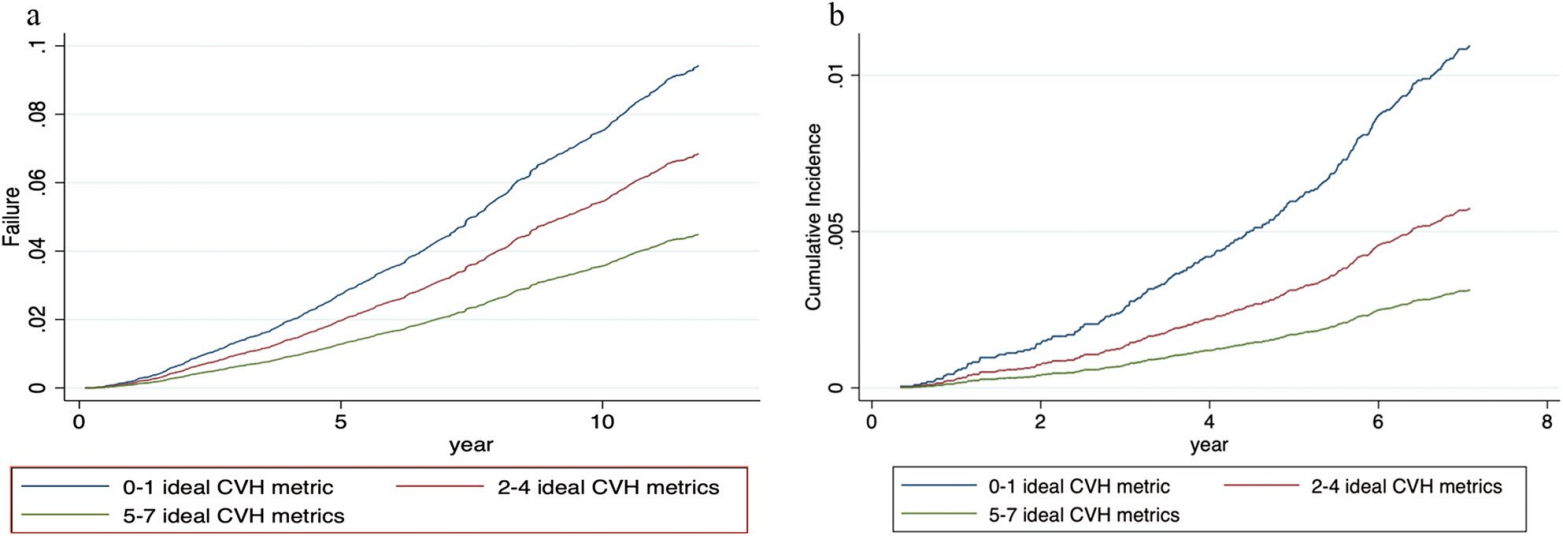
(**a**) Cumulative function curve for all-cause mortality by ideal CVH metrics (blue line: 0–1; orange: 2–4, and green: 5–7 ideal CVH metrics). (**b**) Cumulative function curve for CVD mortality by ideal CVH metrics (blue line: 0–1; orange: 2–4, and green: 5–7 ideal CVH metrics).

Figures 1 and 2 show the cumulative incidence function of the modified CVH metrics (0–7). The highest cumulative incidence of all-cause and CVD mortality was found in individuals with the lowest ideal CVH metric of 0–1, and those with the lowest incidence of all-cause and CVD mortality had ideal CVH metrics of 5–7.

Supplementary Table S1 shows HRs for all-cause and CVD mortality according to each individual’s modified ideal CVH metrics. After controlling for covariates, individuals with ideal levels of most of the CVH metrics were independently associated with lower risks of all-cause and CVD mortality compared with those with poor CVH. In particular, never smoking, HDL concentrations ≥ 60 mg/dL, FPG concentrations < 100 mg/dL, WHtR < 0.5, and BP < 120/80 mmHg were significantly associated with independent lower risks for all-cause and CVD mortality. We did additional analyses to test the associations of BMI metrics (< 23, 23– < 25, and >  = 25 kg/m^2^) and total cholesterol metrics (< 200, 200- < 240, and >  = 240 mg/dL) with the outcomes and there were no significant associations.

## Discussion

This study showed that the prevalence of individual with ideal CVH status (5–7 ideal CVH metrics) was approximately 10% in the participants (5.6% in men and 13.0% in women). This prevalence declined from 10.4% in 2009 to 9.5% in 2014. However, the prevalence of 0–1 ideal CVH metrics was higher in 2014 than in 2009, and only < 0.5% of adult participants achieved all seven ideal CVH metrics in both time periods. We observed a strong dose–response relationship between an increased number of ideal CVH metrics and a lower risk of all-cause and CVD mortality. Individuals who achieved 5–7 ideal CVH metrics had a 53% lower risk of all-cause mortality and 69% lower risk of CVD mortality, when compared with those with 0-l CVH ideal metric. Similarly, individuals who achieved optimal (9–14) overall CVH score had 47% lower risk of all-cause mortality and 49% lower risk of CVD mortality compared with those with inadequate (0–6) overall CVH score category. Increase in one unit of overall CVH score associated with 12% lower risk of all-cause mortality and 19% lower risk of CVD mortality. To the best of our knowledge, this is the first study to show that the distribution of ideal CVH metrics is inversely associated with all-cause and CVD mortality in the Thai population. Overall, this study suggests that lifestyle and metabolic factors are ideal for the prediction of all-cause mortality and CVD outcomes.

The low prevalence of all seven ideal CVH metrics in this study is consistent with a low prevalence in other populations, such as Chinese (0.2%), Koreans (0.67%), and other populations in Western countries with a rate lower than 1%^14^. However, this prevalence varies and a higher prevalence > 1% of all ideal CVH metrics is likely to be due to differences in the definition of metrics and lifestyles among countries.

We modified three metrics in the present study, namely the WHtR as a proxy for obesity, HDL-C, and fruit and vegetable consumption for dietary intake. In additional analyses, we tested the association of using BMI and total cholesterol category with the outcomes and found no or weak association with all-cause and CVD mortality (results shown in supplement). Our previous study and other international studies also supported that the WHtR was better than BMI in predicting CVD outcome^7,15,16^. In the present study, we found that a WHtR < 0.5 was associated with a decline of 10% for all-cause mortality and 36% for CVD mortality. Several studies have reported that the WHtR is a more sensitive predictor for mortality than BMI^8,15,17,18^. In addition, the Thai Ministry of Public Health has launched the WHtR as a national indicator for the target of an anti-obesity campaign. With regard to HDL-C, HDL-C concentrations > 60 mg/dL were independently associated with lower risks of all-cause and CVD mortality in our study. Consistent with our study, a cohort study reported that HDL-C concentrations were inversely associated with CVD outcomes^9^. Several studies in a meta-analysis showed that an increase in HDL-C concentrations was associated with a lower risk of cardiac events, major cardiovascular events, and all-cause mortality^19^.

With regard to vegetable and fruit consumption, the prevalence of an ideal vegetables and fruits intake decreased from 29.1% in 2009 to 24.8% in 2014. This prevalence was relatively low and the findings was consistent with the low level of global fruit and vegetable intake. The global mean fruit intake was 81 g/day and mean vegetable intake was 208 g/day, and only 17 million adults in 187 countries had intake levels >  = 400 g/day, representing 0.4% of the world adult population^20^. The present study showed that fruit and vegetable consumption > 5 portions/day was associated with an 18% lower risk of all-cause mortality and 22% lower risk of CVD mortality, but this did not reach significance, possible due to the small sample size. We used fruit and vegetable consumption as a healthy diet because a large body of evidence have shown to reduce risk of all-cause mortality and non-communicable diseases^21^. Recently, a meta-analysis study including two prospective cohort studies of nurses’ health study and health professional’s follow-up and 24 cohort studies and other 16 cohort studies in 10 European countries demonstrated that a high intake of fruits and vegetables at five servings each day was associated with a lower mortality risk^22,23^. For Blood pressure, we found that untreated BP < 120/80 mmHg was strongly and independently associated with a 24% and 42% lower risk of all-cause and CVD mortality, respectively. Our findings are relatively consistent with a meta-analysis by Guo and Zhang reporting that the HR of untreated BP (< 120/ < 80 mm Hg) was 0.79 for all-cause mortality and that for CVD mortality was 0.47^24^.

Our finding that ideal CVH was inversely associated with CVD and all-cause mortality in the present study is consistent with other studies^4,25^. A systematic review of prospective studies showed an association of a greater number of ideal CVH metrics with lower CVD and all-cause mortality^26^. A meta-analysis of prospective studies reported that an increased number of ideal CVH metrics reduced the risk of CVD and mortality, with a relative risk of 0.54 for all-cause mortality and 0.30 for CVD mortality^24^. We observed similar associations of ideal CVH with all-cause mortality by sex; however, the associations with CVD mortality by sex were slightly different; future research might be warranted to examine this issue. These studies suggest that ideal CVH metrics could be useful for health care practitioners to communicate with individual patients, care givers, and the public, and could be used as a self-assessment tool and track for guidance in lifestyle modification. Clinicians could use this tool at the point of care share decision making to help patients maintain or improve optimal CVH status^27^. The lack of improvement of ideal CVH in the Thai population between two survey rounds (NHESs IV and V) underscores the need to scale up action to improve ideal CVH health. A population and high-risk approach need to be implemented effectively^4^.

The present study has some strengths and limitations. A strength of this study is that it was a cohort of a representative sample of the Thai population. We were able to obtain objective data for CVH metrics to examine their associations with all-cause and CVD specific mortality. In the analysis, we adjusted for potential confounding factors, such as age, sex, and socioeconomic status, and all risk factors in the multivariable regression. However, there are also some limitations. First, the measurements of behavior and clinical data were measured at baseline, and we did not account for possible changes in these variables during the study period. Second, there were few participants in the NHES V cohort and low numbers for each ideal CVH metric, therefore, the statistical power was small. Third, dietary data regarding the quantity of salt intake and fibers including whole grains were not available. However, we used vegetable and fruit intake as a proxy for a healthy diet, with evidence of its association with a lower mortality rate^20^. Nevertheless, recall bias might have been present because data on physical activity, vegetable intake, and smoking were self-reported. Fourth, at the time when the NHES IV was conducted in 2009, the electronic records were not completely available. Consequently, we were not able to ascertain CVD death in the NHES IV cohort. Fifth, this observational study is still limited and may represent reverse or bi-directional causality. Finally, each CVH metric was unweighted and the counting number of ideal metric or overall score system might be relatively simple and possibly less sensitive to make the inter-individual differences and intra-individual change. Further research might account for weighting and trade-off between the accuracy of assessment and simplicity to use.

In conclusion, the prevalence of individuals with 5–7 ideal CVH metrics was relatively low in the Thai population. Individuals with a higher number of ideal CVH metrics have a lower risk of all-cause mortality and cardiovascular mortality. Health professionals could use this tool to motivate the public and patients to be aware of CVH status assessment and track to improve cardiovascular health.

## Supplementary Information

Supplementary Information.

## Data Availability

The datasets generated and/or analysed during the current study are not publicly available due to the policy of the institutes, but are available from the corresponding author on reasonable request.
