# Supplementary material for: Ideal cardiovascular health and all-cause or cardiovascular mortality in a longitudinal study of the Thai National Health Examination Survey IV and V
Source: Sci Rep. 2023 Feb 16;13:2781. doi: 10.1038/s41598-023-29959-1 (PMC9935621; doi:10.1038/s41598-023-29959-1)
Supplement: Supplementary file 1 — Supplementary Information. [file 41598_2023_29959_MOESM1_ESM.docx]

**Supplementary information**

Table S1 All-cause and CVD mortality rate (per 1000 person-years) and HRs by each CVH metrics in both sexes

|  |  | All-cause mortality (NHESs IV and V cohorts) | | | | CVD mortality (NHES V cohort) | | | | |
| --- | --- | --- | --- | --- | --- | --- | --- | --- | --- | --- |
| CVH metrics | No. of deaths | Person-years | Mortality rate | Age-adjusted HR* (95% CI) | Fully adjusted HR** (95% CI) | No. of deaths | Person-years | Mortality rate | Age-adjusted HR* (95% CI) | Fully adjusted HR** (95% CI) |
| **Smoking** |  |  |  |  |  |  |  |  |  |  |
| Never | 1593 | 180464.30 | 8.8 (8.4, 9.3) | 0.63 (0.49, 0.81) | 0.67 (0.53, 0.84) | 116 | 69523.5 | 1.7 (1.4, 2.0) | 0.47 (0.31, 0.70) | 0.45 (0.29, 0.70) |
| Former | 693 | 37858.77 | 18.3 (17.0, 19.7) | 0.75 (0.65, 0.86) | 0.77 (0.66, 0.90) | 57 | 14954.3 | 3.8 (2.9, 4.9) | 0.77 (0.52, 1.13) | 0.76 (0.50, 1.13) |
| Current | 729 | 50443.14 | 14.5(13.4, 15.5) | 1 | 1 | 48 | 16255.9 | 3.0 (2.2, 3.9) | 1 | 1 |
| **Waist-to-height ratio** |  |  |  |  |  |  |  |  |  |  |
| Ideal (<0.5) | 1072 | 105533.72 | 10.2 (9.6, 10.8) | 0.81 (0.68, 0.96) | 0.87 (0.76, 0.99) | 56 | 33931.1 | 1.7 (1.3, 2.1) | 0.63 (0.43, 0.93) | 0.70 (0.46, 0.98) |
| Intermediate (0.5 to <0.6) | 1425 | 125828.57 | 11.3 (10.7, 11.9) | 0.80 (0.68, 0.95) | 0.85 (0.72, 1.01) | 105 | 49134.6 | 2.1 (1.8, 2.6) | 0.66 (0.48, 0.92) | 0.71 (0.51, 0.99) |
| Poor (≥0.6) | 518 | 37403.90 | 13.9 (12.7, 15.1) | 1 | 1 | 60 | 17667.9 | 3.4 (2.6, 4.4) | 1 | 1 |
| **Vegetables and fruits** |  |  |  |  |  |  |  |  |  |  |
| Ideal | 628 | 71599.14 | 8.8 (8.1, 9.5) | 0.80 (0.65, 0.98) | 0.83 (0.66, 1.03) | 37 | 23918.7 | 1.5 (1.1, 2.1) | 0.75 (0.52, 1.07) | 0.84 (0.58, 1.20) |
| Intermediate | 731 | 73740.02 | 9.9 (9.2, 10.7) | 0.78 (0.66, 0.92) | 0.80 (0.68, 0.94) | 48 | 29703.1 | 1.6 (1.2, 2.1) | 0.69 (0.49, 0.96) | 0.73 (0.52, 1.02) |
| Poor | 1656 | 123427.04 | 13.4 (12.8, 14.1) | 1 | 1 | 136 | 47111.9 | 2.9 (2.4, 3.4) | 1 | 1 |
| **Physical activity** |  |  |  |  |  |  |  |  |  |  |
| Ideal | 218 | 24942.90 | 8.7 (7.6, 10.0) | 0.82 (0.67, 1.01) | 0.87 (0.71, 1.06) | 5 | 5900.9 | 0.8 (0.4, 2.0) | 0.50 (0.20, 1.20) | 0.57 (0.23, 1.39) |
| Intermediate | 325 | 31510.33 | 10.3 (9.2, 11.5) | 0.91 (0.69, 1.20) | 0.97 (0.75, 1.25) | 32 | 13303.9 | 2.4 (1.7, 3.4) | 0.91 (0.63, 1.33) | 0.98 (0.67, 1.43) |
| Poor | 2472 | 212312.97 | 11.6 (11.2, 12.1) | 1 | 1 | 184 | 81528.8 | 2.3 (2.0, 2.6) | 1 | 1 |

CVD, cardiovascular diseases; CVH, cardiovascular health

*Adjusted for age, sex; **adjusted for all variables.

Table S1 (continue) All-cause and CVD mortality rate (per 1000 person-years) and HRs by each CVH metrics in both sexes metrics

|  | All-cause mortality (NHESs IV and V cohorts) | | | | | CVD mortality (NHES V cohort) | | | | |
| --- | --- | --- | --- | --- | --- | --- | --- | --- | --- | --- |
| CVH metrics | No. of deaths | Person-years | Mortality rate | Age-adjusted HR* (95% CI) | Fully adjusted HR** (95% CI) | No. of deaths | Person-years | Mortality rate | Age-adjusted HR* (95% CI) | Fully adjusted HR** (95% CI) |
| **BP (mmHg)** |  |  |  |  |  |  |  |  |  |  |
| (SBP/DBP <120/80, untreated) | 551 | 92007.06 | 6.0 (5.5, 6.5) | 0.75 (0.62, 0.90) | 0.75 (0.62, 0.91) | 31 | 33083.7 | 0.9 (0.7, 1.3) | 0.59 (0.38, 0.90) | 0.62 (0.40, 0.97) |
| Intermediate (SBP 120–129, DBP <80, or treated to goal) | 886 | 92424.74 | 9.6 (9.0, 10.2) | 0.77 (0.65, 0.91) | 0.79 (0.66, 0.95) | 56 | 34310.1 | 1.6 (1.3, 2.1) | 0.63 (0.46, 0.87) | 0.66 (0.48, 0.91) |
| SBP ≥130 or DBP ≥80 | 1578 | 84334.41 | 18.7 (17.8, 19.7) | 1 | 1 | 134 | 33339.9 | 4.0 (3.4, 4.8) | 1 | 1 |
| **HDL-C (mg/dL)** |  |  |  |  |  |  |  |  |  |  |
| Ideal (≥60) | 343 | 42955.38 | 8.0 (7.2, 8.9) | 0.80 (0.65, 0.98) | 0.86 (0.71, 1.04) | 32 | 21027.5 | 1.5 (1.1, 2.2) | 0.70 (0.47, 1.03) | 0.81 (0.55, 1.21) |
| Intermediate (40 to <60) | 1113 | 102562.48 | 10.9 (10.2, 11.5) | 0.86 (0.76, 0.97) | 0.92 10.82, 1.02) | 79 | 36799.6 | 2.1 (1.7, 2.7) | 0.81 (0.60, 1.08) | 0.88 (0.65, 1.18) |
| Poor (<40) | 1559 | 123248.34 | 12.6 (12.0, 13.3) | 1 |  | 110 | 42906.5 | 2.6 (2.1, 3.1) | 1 | 1 |
| **FPG (mg/dL)** |  |  |  |  |  |  |  |  |  |  |
| Ideal <100 | 1884 | 199548.14 | 9.4 (9.0, 9.9) | 0.57 (0.45, 0.73) | 0.58 (0.45, 0.74) | 122 | 70427.0 | 1.7 (1.5, 2.1) | 0.48 (0.35, 0.65) | 0.52 (0.38, 0.71) |
| Intermediate (100–125) | 508 | 39564.97 | 12.8 (11.8, 14.0) | 0.60 (0.46, 0.78) | 0.59 (0.45, 0.79) | 33 | 17870.4 | 1.8 (1.3, 2.6) | 0.39 (0.26, 0.60) | 0.41 (0.27, 0.63) |
| Poor (≥126) | 623 | 29653.10 | 21.0 (19.4, 22.7) | 1 | 1 | 66 | 12436.2 | 5.3 (4.2, 6.8) | 1 | 1 |
| Additional analysis | | |  |  |  |  |  |  |  |  |
| BMI kg/m^2^ |  |  |  |  |  |  |  |  |  |  |
| Ideal (<23) | 1457 | 103224.53 | 14.1 (13.4, 14.9) | 1.17 (1.02, 1.35) | 1.22 (1.10, 1.35) | 85 | 36024.36 | 2.4 (1.9, 29) | 0.94 (0.70, 1.27) | 0.98 (0.70, 1.37) |
| Intermediate (23-<25) | 558 | 54064.79 | 10.3 (9.5, 11.2) | 0.94 (0.79, 1.11) | 0.98 (0.82, 1.16) | 52 | 20035.93 | 2.6 (2.0, 3.4) | 1.11 (0.80, 1.55) | 1.21 (0.85, 1.72) |
| Poor (>=25) | 1000 | 111476.87 | 9.0 (8.4, 9.5) | 1 | 1 | 84 | 44673.38 | 1.9 (1.5, 2.3) | 1 | 1 |
| Total cholesterol (mg/dL) |  |  |  |  |  |  |  |  |  |  |
| Ideal (<200) | 1309 | 115823.55 | 11.3 (10.7, 11.9) | 1.08 (0.  88, 1.34) | 1.05 (0.86, 1.28) | 103 | 48316.22 | 2.1 (1.8, 2.6) | 1.06 (0.75, 1.50) | 0.92 (0.63, 1.34) |
| Intermediate (200-<240) | 957 | 88752.77 | 10.8 (10.1, 11.5) | 1.02 (0.85, 1.22) | 1.02 (0.86, 1.20) | 75 | 31295.17 | 2.4 (1.9, 3.0) | 1.19 (0.83, 1.72) | 1.21 (0.83, 1.76) |
| Poor (>=240) | 749 | 64189.88 | 11.7 (10.9, 12.5) | 1 | 1 | 43 | 21122.28 | 2.0 (1.5, 2.7) | 1 | 1 |

CVD, cardiovascular diseases; CVH, cardiovascular health

*Adjusted for age, sex; **adjusted for all age, sex, education, area of residence, alcohol drink and dyslipidemia medication.

Table S2 All-cause and CVD mortality rate (per 1000 person-years) and HRs by ideal CVH in men

|  | All-cause | | | | | CVD | | | | |
| --- | --- | --- | --- | --- | --- | --- | --- | --- | --- | --- |
| Number of ideal CVH metrics | No. of deaths | Person-years | Mortality rate | Age-adjusted HR* (95% CI) | Fully adjusted HR** (95% CI) | No. of deaths | Person-years | Mortality rate | Age-adjusted HR* (95% CI) | Fully adjusted HR** (95% CI) |
| 0–1 | 642 | 31723.81 | 20.2 (18.7, 21.9) | 0.77 (0.52, 1.13) | 0.76 (0.52, 1.11) | 51 | 12916.24 | 3.9 (3.0, 5.2) | 0.79 (0.52, 1.22) | 0.79 (0.51, 1.21) |
| 2 | 522 | 35176.59 | 14.8 (13.6, 16.2) | 0.78 (0.61, 1.0) | 0.78 (0.61, 1.0) | 36 | 12864.72 | 2.8 (2.0, 3.9) | 0.67 (0.39, 1.14) | 0.68 (0.40, 1.16) |
| 3 | 381 | 30084.29 | 12.7 (11.4, 14.0) | 0.60 (0.36, 0.99) | 0.60 (0.36, 1.00) | 19 | 9383.80 | 2.0 (1.3, 3.2) | 0.54 (0.21, 1.37) | 0.55 (0.22, 1.41) |
| 4 | 127 | 15163.36 | 8.4 (7.0, 10.0) | 0.46 (0.25, 0.83) | 0.48 (0.26, 0.88) | 5 | 4179.01 | 1.2 (0.5, 2.9) | 0.39 (0.05, 2.96) | 0.41 (0.05, 3.11) |
| 5–7 | 24 | 5388.46 | 4.5 (3.0, 6.6) | 1 | 1 | 1 | 1541.37 | 0.6 (0.1, 4.6) | 1 | 1 |
| Total | 1696 | 117536.50 | 14.4 (13.8, 15.1) |  |  | 112 | 40885.14 | 2.7 (2.3, 3.3) |  |  |
| Overall CVH score | |  |  |  |  |  |  |  |  |  |
| Inadequate (0-6) | 1014 | 50811.03 | 20.0 (18.8, 21.2) | 1 | 1 | 80 | 19639.19 | 4.1 (3.3, 5.1) | 1 | 1 |
| Average (7-8) | 488 | 40620.17 | 12.0 (11.0, 13.1) | 0.66 (0.53, 0.83) | 0.67 (0.53, 0.83) | 24 | 13704.28 | 1.8 (1.2, 2.6) | 0.51 (0.32, 0.81) | 0.52 (0.33, 0.81) |
| Optimal (9-14) | 194 | 26105.31 | 7.4 (6.5, 8.6) | 0.50 (0.34, 0.72) | 0.52 (0.35, 0.75) | 8 | 7541.66 | 1.1 (0.5, 2.1) | 0.42 (0.20, 0.88) | 0.43 (0.21, 0.91) |
| Overall CVH score increase per unit) | 1696 |  |  | 0.89 (0.84, 0.94) | 0.89 (0.84, 0.94) | 112 |  |  | 0.86 (0.78, 0.94) | 0.86 (0.79, 0.95) |

*Adjusted for age and sex; ** adjusted for age, sex, education, urban/rural area, and alcohol drinking.

Table S3 All-cause and cardiovascular mortality rate (per 1000 person-years) and HRs by ideal cardiovascular health in women

|  | All-cause | | | | | CVD | | | | |
| --- | --- | --- | --- | --- | --- | --- | --- | --- | --- | --- |
| Number of ideal CVH metrics | No. of deaths | Person-years | Mortality rate | Age-adjusted HR* (95% CI) | Fully adjusted HR** (95% CI) | No. of deaths | Person-years | Mortality rate | Age-adjusted HR* (95% CI) | Fully adjusted HR** (95% CI) |
| 0–1 | 346 | 19268.36 | 18.0 (16.2, 19.9) | 0.72 (0.59, 0.88) | 0.71 (0.58, 0.87) | 50 | 8888.28 | 5.6 (4.3, 7.4) | 0.35 (0.22, 0.56) | 0.35 (0.22, 0.56) |
| 2 | 465 | 40713.01 | 11.4 (10.4, 12.5) | 0.62 (0.47, 0.81) | 0.61 (0.47, 0.78) | 27 | 16786.14 | 1.6 (1.1, 2.3) | 0.39 (0.23, 0.65) | 0.39 (0.23, 0.67) |
| 3 | 315 | 42411.35 | 7.4 (6.6, 8.3) | 0.61 (0.46, 0.80) | 0.60 (0.44, 0.79) | 19 | 16649.57 | 1.1 (0.7, 1.8) | 0.45 (0.23, 0.86) | 0.48 (0.25, 0.92) |
| 4 | 150 | 30880.97 | 4.9 (4.1, 5.7) | 0.46 (0.30, 0.731 | 0.46 (0.29, 0.76) | 11 | 11146.27 | 1.0 (0.5, 1.8) | 0.25 (0.06, 1.11) | 0.29 (0.06, 1.32) |
| 5–7 | 43 | 17956.00 | 2.4 (1.8, 3.2) | 1 | 1 | 2 | 6378.26 | 0.3 (0.1, 1.3) | 1 |  |
| Total | 1319 | 151229.70 | 8.7 (8.3, 9.2) |  |  | 109 | 59848.52 | 1.8 (1.5, 2.2) |  |  |
| Overall CVH score |  |  |  |  |  |  |  |  |  |  |
| Inadequate (0-6) | 760 | 50663.94 | 15.0 (14.0, 16.1) | 0.76 (0.63, 0.92) | 0.76 (0.63, 0.91) | 75 | 21390.90 | 3.5 (2.8, 4.4) | 0.54 (0.34, 0.87 | 0.58 (0.36, 0.94) |
| average (7-8) | 388 | 50351.41 | 7.7 (7.0, 8.5) | 0.58 (0.46, 0.73) | 0.58 (0.45, 0.75) | 21 | 19658.70 | 1.1 (0.7, 1.6) | 0.56 (0.30, 1.02) | 0.66 (0.35, 1.22) |
| Optimal (9-14) | 171 | 50214.35 | 3.4 (2.9, 4.0) | 1 | 1 | 13 | 18798.92 | 0.7 (0.4, 1.2) | 1 | 1 |
| Overall CVH score increase per unit) | 1319 |  |  | 0.87 (0.83, 0.91) | 0.87 (0.84, 0.92) | 109 |  |  | 0.76 (0.67, 0.85) | 0.76 (0.67, 0.86) |

*Adjusted for age and sex; ** adjusted for age, sex, education, urban/rural area, and alcohol drinking and dyslipidemia medication.

**Flow chart of participants included**

20,448 participants enrolled in the 2009 survey and 19,476 participants in the 2014 survey

Participants remained (n= 16,439) in 2009 and (n= 15,587) in 2014

Excluding participants aged <20 years old

29,718 participants included in the final analysis for all-cause mortality and 14,499 participants for CVD mortality analysis (15,219 participants in 2009 and 14,499 in 2014)

Excluding participants who missed data on CVH parameters

Excluding participants with a BMI <18.5 kg/m^2^, a history of a CVD, or chronic kidney disease

Participants aged 20 and older in the 2009 survey (n=19,337) and the 2014 survey (n = 18,126)
